# Synthesis of a Cytokinin Linked by a Spacer to Dexamethasone and Biotin: Conjugates to Detect Cytokinin-Binding Proteins

**DOI:** 10.3390/molecules21050576

**Published:** 2016-04-30

**Authors:** You Wang, David S. Letham, Peter C. L. John, Ren Zhang

**Affiliations:** 1 School of Biological Sciences, University of Wollongong, Wollongong, NSW 2522, Australia; wyou@uoguelph.ca; 2 Research School of Biology, Australian National University, Canberra, ACT 0200, Australia; david.letham@bigpond.com (D.S.L.); peteandjenny.john@gmail.com (P.C.L.J.)

**Keywords:** 6-benzylaminopurine (BAP), BAP-dexamethasone conjugates, BAP-biotin conjugates, yeast three hybrid, cytokinin-binding proteins

## Abstract

Yeast cells expressing cDNA libraries have provided two new approaches to facilitate further identification of cytokinin-binding proteins and receptors. These are the yeast three hybrid (Y3H) system and fluorescence activated cell sorting (FACS). The Y3H system requires a synthetic hybrid ligand comprising an “anchor” moiety (e.g., dexamethasone) linked to a cytokinin via a spacer. In the yeast nucleus, this ligand by binding connects two fusion proteins leading to a reporter gene activation and detection and characterisation of cytokinin binding proteins. Herein is reported the first synthesis of dexamethasone-cytokinin ligands with a spacer linkage. This was attached to the purine ring of 6-benzylaminopurine (BAP) at positions 2, 8 or 9. To achieve this, dexamethasone was modified by periodate oxidation yielding a carboxylic group used for conjugation to the spacer by amide formation. Biotinyl derivatives of cytokinins for FACS included those synthesised by reaction of an activated ester of biotin with 8-(10-amino-decylamino) derivatives of BAP and BAP 9-riboside. Properties of the conjugates and some biological situations where they could be applicable are discussed briefly.

## 1. Introduction

The phytohormones termed cytokinins regulate numerous aspects of plant development including cell division, leaf senescence, apical dominance and nutrient translocation, and appear to be essential for plant growth. Cytokinins are *N*^6^-substituted adenines and the naturally occurring compounds are of two types: compounds with an isoprenoid substituent (e.g., zeatin, 6-(4-hydroxy-3-methylbut-2-enylamino)purine) and those with an *N*^6^-aromatic ring (e.g., 6-benzylaminopurine (BAP)) [1]. Three membrane-located histidine kinase cytokinin receptors, termed AHK2, AHK3 and AHK4, have been characterised in *Arabidopsis thaliana* [2] and are located on the plasma membrane and/or endoplasmic reticulum [3]. Similar receptors necessary for nodule organogenesis have been detected in roots of *Lotus japonicas* plants [4]. Studies of binding of cytokinin-active BAP derivatives to the histidine kinase receptors indicated that another receptor able to bind BAP derivatives occurs in plants and it was proposed that diverse recognition systems may operate for cytokinins [5]. 

In addition to the membrane-bound histidine kinase receptors, numerous cytokinin-binding soluble proteins (CBPs) have been detected in higher plants [6,7]. Most have been partially characterised and some exhibit receptor-like properties including a correlation between affinities for different cytokinins and their biological activities. However, in no case has the encoding gene for the CBP been identified and cloned and the physiological significance of the CBPs remains uncertain. The very few which do appear to exert a regulatory role include 67 kDa CBPs from barley and *Arabidopsis* leaves. These proteins bind to zeatin to promote *in vitro* transcription directed by RNA polymerase I and also appear to be nuclear CBPs [8,9]. Further significant CBPs are a 70 kDa protein involved in cytokinin regulation of transcription in plastids [10] and HOG1 protein of *Arabidopsis* which appears to play a role in modulating cytokinin signal transduction [11]. It is also possible that some CBPs may have a passive role by simply sequestering cytokinin to stabilise and protect the hormone.

In addition to their role as plant hormones, cytokinins may influence the normal growth and activity of some human cells *in vitro* [12,13,14,15]. Kinetin (6-furfurylaminopurine) suppressed free radical formation associated with platelet aggregation and appeared to have antithrombotic activity *in vivo* [16]. Considerable interest now focuses on the ability of cytokinin 9-ribosides to inhibit growth of cancer cells and there are many reports in this regard, including some in the early cytokinin literature, e.g., [17,18]. While cytokinin bases induced differentiation of human leukaemia HL-60 cells into granulocytes [19], cytokinin ribosides caused apoptosis in these cells [20] and in HeLa and mouse melanoma cells [21]. While the mass of melanoma tumours on mice was very markedly reduced by injection of kinetin riboside [21], this riboside also induced G2/M arrest and death in human heptamoa cells *in vitro* [22] and inhibited cyclin expression in myeloma cells causing cell cycle arrest [23]. In a recent study, the cytotoxic activities of almost all known naturally occurring cytokinins (over 40 compounds) were determined towards a diverse range of human tumour cell lines [24,25]. Cytotoxicity and cytokinin activity in plant cell division bioassays did not correlate. iPA and the ribosides of kinetin, BAP and *o*-hydroxy-BAP (*o*-topolin) were the most effective inhibitors of tumour growth, exhibiting cytotoxicity at about 0.5–5 μM in 7 of 9 tumour cell lines. Small structural changes in the *N*^6^-substituent altered inhibitory activity markedly, suggesting the receptors/binding proteins involved recognised this structural feature on which growth promotion in plants also depends. 

Cytokinins may also be important in the development of insects. Larvae have been found to synthesise isoprenoid cytokinins, which are secreted to induce gall formation [26]. In newly laid eggs of the insect *Locusta migratoria*, the cytokinin *N*^6^-isopentenyl adenosine 5′-monophosphate occurs in ester linkage to the steroid hormone ecdysone [27]. Important contributions from cytokinins are suggested by the capacity of BAP at less than 10^−6^ M in culture medium to replace foetal calf serum in stimulating growth of *Drosophila* cells [28] and the ability of kinetin added at low levels to the diet of fruit flies (*Zaprionus paravittiger*) to prolong their life span [29].

Further analysis of cytokinin action at the molecular level in higher plants, mammalian cells and insects requires characterisation of cytokinin receptors/binding proteins in diverse organisms, a difficult biochemical problem. Two new approaches that could be developed for this task are based on yeast cells expressing cDNA libraries from target organisms. One is based on the yeast three hybrid (Y3H) system [30] and the other on the fluorescence activated cell sorting technique [31,32]. The proposed former system is designed on the detection of a specific reporter gene signal triggered by the interaction between DNA-binding and activation domains of the yeast GAL4 transcription factor upon cytokinin binding to a receptor in yeast cells. It would utilise three key components, namely, (1) a fusion protein with a dexamethasone receptor linked to the DNA-binding domain of GAL4; (2) a second fusion protein in which a target CBP expressed by the cDNA library is fused to the activation domain of GAL4; and (3) a synthetic hybrid ligand in which dexamethasone (regarded as the ‘anchor’ molecule [30]) is linked to BAP through a C_10_ spacer. Once the hybrid ligand is bound by both dexamethasone and BAP receptors produced in yeast, the respectively fused DNA-binding and activation domains will join together to trigger the expression of the reporter gene controlled by GAL4. This allows selection of yeast cells that harbour the cytokinin-binding protein. The methodology for producing the two fusion proteins in yeast has been established [30] and only the ligand molecule requires development to make the system relevant to CBPs. This paper therefore details the first synthesis of compounds in which a cytokinin is linked at one of three positions through a spacer of 10 carbon atoms to a dexamethasone moiety. In some studies unrelated to cytokinins, this has been replaced by alternative “anchor” molecules, e.g., methotrexate and FK506 [30]. 

Since individual yeast cells, in a population that has been exposed to a gene library, receives only a single introduced gene for expression, this can be readily recovered and the encoded protein characterised. The Y3H method is a powerful system and has been used to identify the targets of several inhibitors and drugs in mammalian cells and target proteins for cucurbic acid methyl ester and for salicylic acid in plant cell libraries [30]. This system has advantages over earlier biochemical strategies in that the interaction between receptor and ligand is determined *in vivo*. Furthermore the method is independent of the quantity of biological material available and can lead directly to the sequencing and identification of hormone-binding protein.

However, the Y3H method as currently employed is limited to the identification of soluble proteins that move to the yeast nucleus, while proteins located on membranes and organelles will not be recognised. To test for such proteins, we here also show that cytokinins can be chemically modified to conjugates that retain biological properties of cytokinin but are also targets for fluorescence detection. With such reagents, fluorescence activated cell sorting (FACS) [31,32] can be used to recover individual yeast cells that express a membrane-bound CBP, as discussed below. 

This system utilising FACS is based on BAP-biotin conjugates. If any yeast cell expresses a membrane-bound CBP, FACS would be able to recognise it due to binding BAP-biotin conjugate detectable by streptavidin-phycoerithrin. The sorted cell would be recovered by growth on agar medium leading to characterisation of the introduced gene for a cytokinin-binding protein and study of its cellular location by fluorescent microscopy. Hence, in this paper we report the synthesis and properties of compounds in which BAP is linked to either dexamethasone or biotin through a C_1__0_ spacer moiety for use in the Y3H and FACS methods respectively. 

## 2. Results and Discussion

### 2.1. Synthesis of Dexamethasone Conjugates

**Figure 1:**
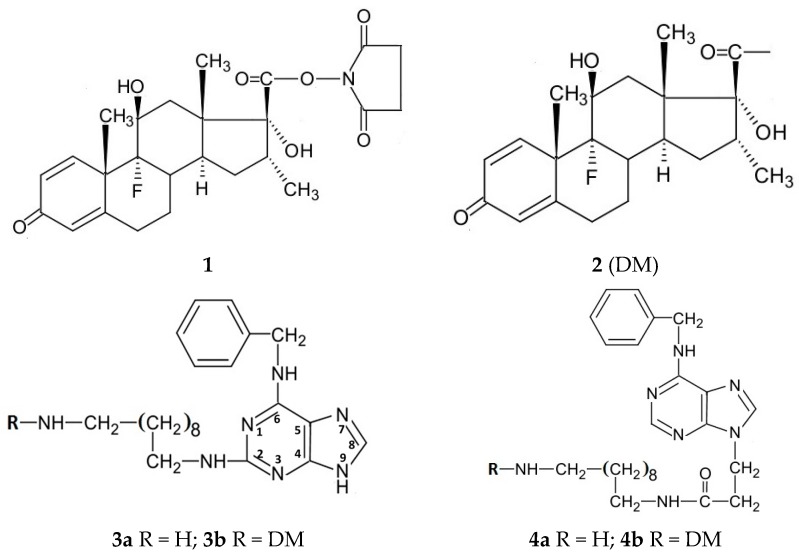

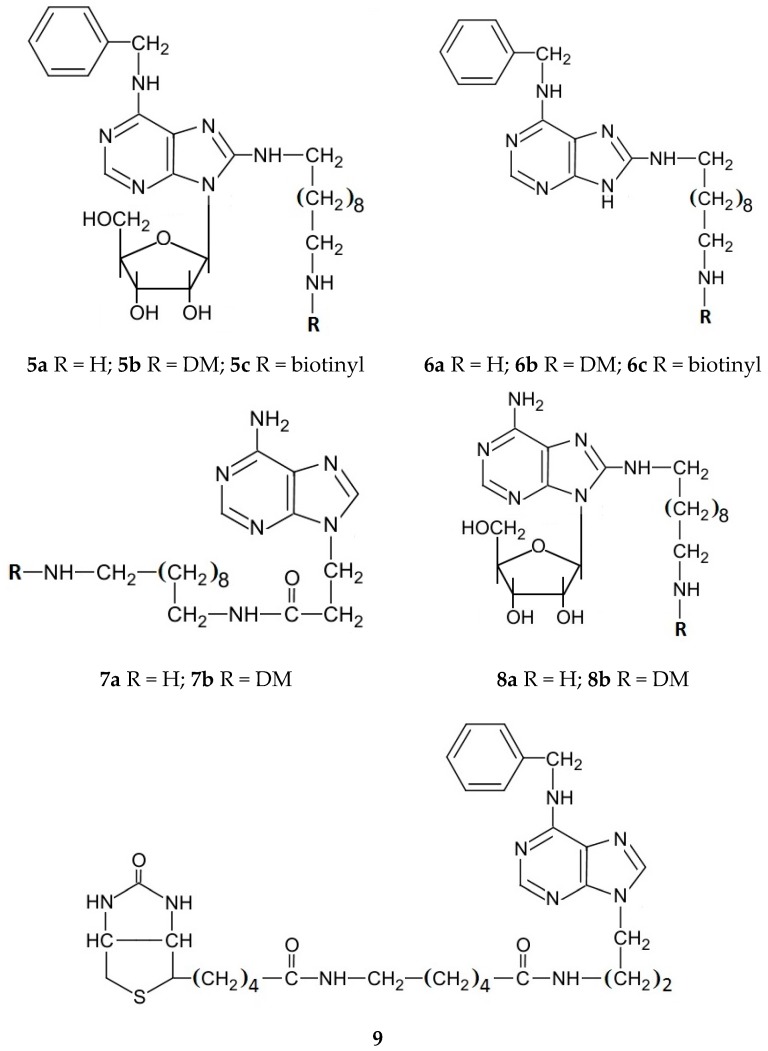
Structures of compounds used and synthesised in this study.

Dexamethasone does not have a functional group suitable for conjugation to a cytokinin moiety. To confer such a group, dexamethasone was oxidised with periodate to convert the hydroxymethyl ketone moiety into a carboxyl group and the resulting compound is referred to herein as “dexamethasone acid”. Conversion to a succinimidyl ester (**1**; Figure 1) by reaction with *N*-hydroxy-succinimide in the presence of dicyclohexylcarbodiimide yielded an activated ester. This reacted at 20–25 °C with the free amino group of amino-alkyl amino adenines to yield the corresponding amides with a dexamethasyl (DM) moiety (**2**). This approach based on activated ester and then amide formation appears to be the only convenient conjugation procedure for dexamethasone.

**Figure 2:**
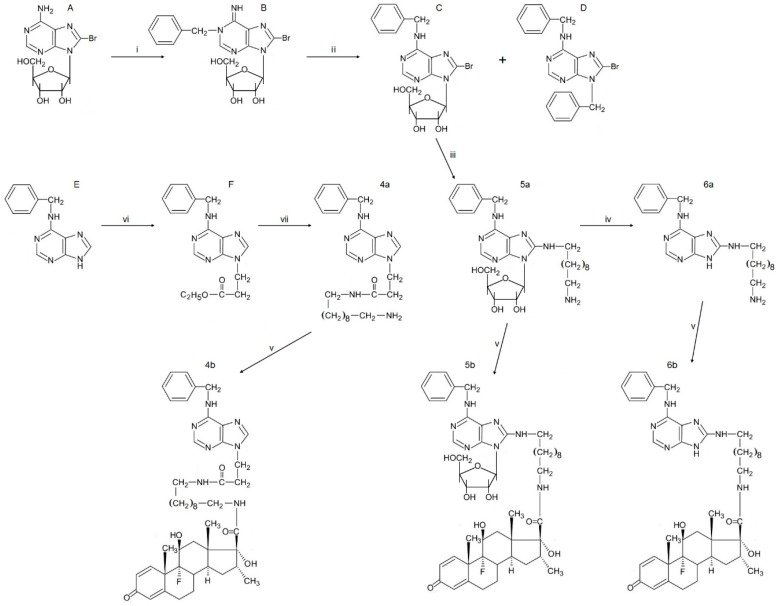
The sequence of reactions used to synthesise BAP-dexamethasone conjugates with purine ring linkage at C-8 (**5b**, **6b**) or N-9 (**4b**). (A) 8-bromoadenosine; (B) 1-benzyl-8-bromoadenosine; (C) 6-benzylamino-8-bromopurine 9-riboside; (D) 6-benzylamino-9-benzyl-8-bromopurine; (E) 6-benzylaminopurin; (F) ethyl 3-(6-benzylaminopurine-9-yl)propionate. The reactions are: (i) benzylbromide in DMF at 37 °C; (ii) 0.25 M NH_4_OH at 37 °C; (iii) 1,10-diaminodecan in *n*-PrOH at 100 °C; (iv) methanolysis with HCl at 37 °C; (v) activated ester of “dexamethasone acid” at 23 °C; (vi) ethyl acrylate/NaOEt; (vii) 1,10-diaminodecane in toluene at 110 °C.

The amine 2-(10-aminodecylamino)-6-benzylaminopurine (**3a**) was readily prepared by reacting 1,10-diaminodecane with 6-benzylamino-2-chloropurine at 156 °C in isoamyl alcohol. However, the synthesis of N-9 alkyl derivatives of BAP and adenine terminating in a free amino group was more problematic, but was achieved after initial reaction with ethyl acrylate in ethanol containing sodium ethoxide (a known Michael type reaction at N-9; Figure 2). This introduced a CH_2_CH_2_COOC_2_H_5_ moiety at N-9 of adenine and BAP and further reaction with 1,10-diaminodecane at 110 °C yielded the amines 3-(6-benzylaminopurin-9-yl)propionamido-decylamine (**4a**) and 3-(6-aminopurin-9-yl)propionamido-decylamine (**7a**).

To facilitate synthesis of BAP linked at position C-8 to an aminoalkyl moiety, 8-bromoadenosine was benzylated with benzyl bromide (Figure 2). Alkylation of a 9-substituted adenine is known [33] to yield a 1-substituted derivative and a 1-benzyl product was obtained above. This was then converted by the Dimroth rearrangement [34] to 6-benzylamino-8-bromopurine 9-riboside. The latter was separated from 6-benzylamino-9-benzyl-8-bromopurine, which also formed in the benzylation reaction. The benzylamino riboside and 8-bromo-adenosine were then reacted with 1,10-diamino-decane yielding 8-(10-aminodecylamino)-6-benzylamino-9-β-d-ribofuranosylpurine (**5a**) and 6-amino-8-(10-aminodecylamino)-9-β-d-ribofuranosylpurine (**8a**). The ribose moiety of **5a** was cleaved at 37 °C in methanol containing HCl and 2,2-dimethoxypropane yielding **6a**.

The above 6 amines (**3a**–**8a**) reacted readily with the dexamethasone active ester (**1**) yielding the corresponding amide conjugates (**3b**–**8b**) with the dexamethasyl moiety (DM, **2**). These compounds were characterised by UV and mass spectral data (Table 1). The mass spectral data confirm the structures assigned to compounds **3b** to **8b**. Further confirmation was provided by the analysis of their UV spectra, which shows that each contains a dexamethasone acid moiety linked to BAP (**3b**–**6b**) or adenine (**7b, 8b**). Both dexamethasone acid and the purine moieties in the conjugates exhibit strong UV absorption but the λ_max_ values for the two are quite different (dexamethasone acid, λ_max_ 240 nm; the substituted purines typically show absorption at 200–240 nm and at 265–300 nm but the spectrum depends on the site of substitution (*i.e.*, linkage). Pure crystalline model purine compounds were synthesised for UV spectral comparison with the conjugates. Each synthesised compound and the purine part of the corresponding conjugate would exhibit an identical UV spectrum. These compounds were 6-benzylamino-2-*n*-hexylaminopurine (*cf.* **3b**), ethyl 3-(6-benzylaminopurin-9-yl)propionate (*cf.* **4b**), 6-benzylamino-8-*n*-hexylaminopurine (*cf.* **6b**), ethyl 3-(6-aminopurin-9-yl)propionate (*cf.* **7b**) and 8-*n*-pentylaminoadenosine (*cf.* **8b**). The UV spectrum of each compound differed considerably from that of the corresponding conjugate in Table 1, but when dexamethasone acid was added in an equimolar amount to the model compound solution, the spectrum became identical to that of the conjugate. This confirms the purity and identity of the conjugates listed in Table 1.

### 2.2. Synthesis of Biotin Conjugates

To synthesise the biotin-cytokinin conjugates **5c** and **6c** (Figure 1) for FACS, biotin was converted to the succinimidyl ester and reacted with the amines **5a** and **6a** respectively. Both **5c** and **6c** contain a biotin moiety linked through a C_10_ spacer to the C-8 of the purine ring, but **5c** has at N-9 a ribofuranosyl moiety, a frequent feature of naturally occurring cytokinins. The biotin structure does not exhibit significant UV absorption and the UV spectrum of the biotin conjugates closely match those of the corresponding BAP-amines used in their synthesis.

In order to prepare biotin conjugates, suitable for FACS, from purines with short amino- alkyl sides chains, an amidocaproyl spacer moiety was added to the C_4_ side chain of biotin. Formation of the succinimidyl active ester and reaction with, for example, 9-(3-aminopropyl)-6-benzylaminopurine yielded **9** with a nine carbon linkage between the biotin and N-9 of BAP. Addition of a second spacer moiety would yield a biotin derivative to which BAP could be conjugated directly through an amino group inserted on the benzene ring.

### 2.3. Cytokinin Activity of Conjugates

The activities of the dexamethasone- and biotin-BAP conjugates were determined using a bioassay based on the ability of cytokinins to induce betacyanin pigment formation in the excised cotyledons of *Amaranthus* seedlings when cultured in darkness [35,36]. The bioassay shows high sensitivity and specificity for cytokinins. 

The dexamethasone conjugates **3b**–**6b** all exhibited cytokinin activity when tested alone, but this was much less than that of BAP. They also significantly/ reduced the activity of BAP and **6b** was the most active (Table 2). These results suggest that the dexamethasone conjugates possess certain low cytokinin activity but can compete with BAP for receptor binding. The 6-aminopurine-dexamethasone conjugates **7b** and **8b** (data not shown) were inactive indicating that the response observed for **3b**–**6b** required the benzyl substituent. Substitution of the 6-amino group is required for cytokinin activity in plants. The biotin-BAP conjugates **5c** and **6c** exhibited very similar activity to that of the corresponding dexamethasone compounds **5b** and **6b** (data not shown).

The activity of BAP in the presence of compounds **3b**–**6b** is particularly relevant. Compounds **3b**–**6b** (Table 2) and also **5c** and **6c** (data not shown) reduced the response observed for BAP, **6b** with a purine C-8 linkage being the most effective. This is consistent with the view that the above conjugates compete with BAP for binding at a receptor. Hence the conjugates reported above, especially **6b**, appear to be suitable for a Y3H system for cytokinin receptors in plants.

Unlike BAP, the above conjugates (**3b**–**6b**) do not appear to function effectively after binding to the receptor resulting in reduced response in the bioassay. It is relevant that cytokinin analogues are now recognised that bind to characterised cytokinin receptors competing with active cytokinins, but the analogues evoke a negligible cytokinin response [37,38,39].

Relevant results were obtained with three simple alkyl derivatives of BAP. The BAP derivatives 2-hexylamino-BAP (A), 9-undecenyl-BAP (B) and 8-hexylamino-BAP (C) which resemble **3b**, **4b** and **6b** respectively, but lack a dexamethasone moiety, showed cytokinin activity very similar to that of the above three dexamethasone conjugates (data not shown). Hence the reduced cytokinin activity of **3b**–**6b**, relative to that of BAP, is probably largely attributable to the long chain alkyl substituents present. While the compounds A, B and C all reduced the activity of BAP in the bioassay, C with a C-8 substituent appeared to be the most effective. The increment in betacyanin absorbance induced by BAP (5 μM) was reduced by A, B and C (each at 20 μM) by 23%, 15% and 39% respectively. Based on this result and the data of Table 2, it can be concluded that C-8 of BAP is the preferable linkage position for synthesis of conjugates for cytokinin binding studies. 

Finally, it should be noted that the betacyanin response was used as a convenient bioassay to characterise the synthesised conjugates, but the relevant receptor is probably one of the identified membrane-associated receptors (compare [37,39]). The proposed Y3H system is very unlikely to detect this receptor or similar membrane-bound receptors that cannot translocate to the yeast nucleus. Soluble non-membrane bound CBPs have been detected in plants [8,9]. The proposed Y3H system has the potential to identify and clone these soluble proteins and thus define new regulatory CBPs in plants.

## 3. Experimental Section

### 3.1. Thin-Layer Chromatography (TLC)

Merck silica gel 60 PF_254_ was used for normal phase TLC and layers were developed in tanks lined with filter paper saturated with solvent. Solvents used were (proportions are by vol.): (A) chloroform/methanol (9:1); (B) chloroform/methanol (9:1) with 14 M NH_4_OH added to solvent in tank (0.2 mL/100 mL) and to paper lining tank (several drops); (C) chloroform/methanol (4:1); (D) chloroform/methanol (4:1) plus NH_4_OH as for solvent B; (E) *n*-butanol/H_2_O/14 M NH_4_OH (6:2:1) upper phase; (F) methyl acetate/H_2_O/acetic acid (500:125:40). Products were eluted with the minimum volume of ethanol (unless stated otherwise) after the silica gel had been packed into a small column. 

### 3.2. UV and Mass Spectra

UV spectra were recorded in ethanol–H_2_O (95:5 *v*/*v*) containing 0.1 M HCI (acid spectra) or 0.2 M NH_4_OH (base spectra) using a Shimadzu UV-240 spectrophotometer. Electron-impact mass spectra (EI-MS) were determined at 70 eV and samples were introduced via the direct probe inlet. A VG ZAB/2F mass spectrometer was used to determine fast atom bombardment mass spectra (FAB-MS) at 8 kV using a glycerol matrix. For electrospray ionization spectra (ESI-MS), the samples were introduced by direct infusion in a methanol solution (0.3–0.6 mM) for positive ion spectra. Molecular formulae were determined by high resolution (HR) ESI-MS, with a Waters Xevo G1 QTof mass spectrometer using the following parameters: capillary voltage, 2.5 kV; source temperature, 80 °C; sampling cone voltage, 20 V; desolvation temperature, 350 °C.

### 3.3. Synthesis of Aminoalkylaminopuries

#### 3.3.1. 2-(10-Aminodecylamino)-6-benzylaminopurine (**3a**)

2,6-Dichloropurine was reacted with benzylamine to yield 6-benzylamino-2-chloropurine. This compound (21 mg) and 1,10-diaminodecane (46 mg) were suspended in isoamyl alcohol (0.75 mL) and the mixture was heated at 156 °C for 8 h. The reaction solution was diluted with water, acidified with acetic acid and extracted with *n*-butanol/ethylacetate (1:1 by vol). The extracts were concentrated and subjected to preparative TLC on silica gel (solvent D) yielding compound **3a**. UV (acid): λ_max_ (log ε) 237 (4.30), 252 (4.32), 291 (4.20) nm; λ_min_ (log ε) 242 (4.29), 271 (3.94) nm. UV (base): λ_max_ (log ε) 232 (4.36), 255 (sh) (3.96), 291 (4.03) nm; λ_min_ (log ε) 268 (3.72) nm. EI-MS *m*/*z* (rel. int.) main ions: 395 (M^+^, 57%), 267 (21), 253 (54), 240 (37), 106 (18), 91 (100). 

#### 3.3.2. 3-(6-Benzylaminopurin-9-yl)propionamido-decylamine (**4a**)

BAP was reacted with ethyl acrylate to yield ethyl 3-(6-benzylaminopurin-9-yl)propionate [39]. This ester with MS, NMR and other properties identical to those previously reported [36] (102 mg), 1,10-diaminodecane (163 mg) and toluene (200 μL) were heated in a sealed vial at 110 °C for 2.5 h. The reaction mixture was dissolved in ethanol (2 mL) and water (4 mL) added. The solution was acidified with HCl and extracted with ethyl acetate (extracts discarded). The aqueous phase was then adjusted to pH 11–12 with KOH and extracted again with ethyl acetate. The extracts were washed with water, concentrated and subjected to preparative TLC on silica gel (solvent D) yielding compound **4a**. UV (acid): λ_max_ (log ε) 268 (4.23) nm; λ_min_ (log ε) 237 (3.47) nm. UV (base): λ_max_ 271 nm; λ_min_ 233 nm. EI-MS *m*/*z* main ions: 451 (M^+^, 70%), 253 (24), 252 (19), 225 (100), 224 (49), 106 (82), 91 (90). 

#### 3.3.3. 8-(10-Aminodecylamino)-6-benzylamino-9-β-d-ribofuranosylpurine (**5a**)

6-Benzylamino-8-bromopurine 9-riboside was first prepared as an intermediate in the synthesis of **5a**. In an initial reaction, benzylbromide (130 μmol) was added to 8-bromoadenosine (22 μmol) dissolved in dry DMF (250 μL) containing triethylamine (7 μL) and the mixture was kept at 37 °C for 3 days. After addition of further triethylamine (10 μL), the solvent was evaporated and the residue dissolved in 50% ethanol (0.5 mL) to which 0.5 M aqueous NH_4_OH (0.5 mL) was added. The solution was kept at 37 °C for 24 h to induce rearrangement of the N^1^-benzyl product (identified by characteristic UV spectrum [33]) to the *N*^6^ compound. Preparative TLC (solvent E) yielded two main products: A (*R*_F_ 0.45) and B (*R*_F_ 0.83) together with some unreacted bromoadenosine (*R*_F_ 0.40). Compound A (identified as *6-benzylamino-8-bromopurine 9-riboside*): UV (100% ethanol) λ_max_ 273 nm, λ_min_ 238 nm; EI-MS *m*/*z* 435/437 (M^+^, 1.2%), 405/407 (1.4), 346/348 (11), 332/334 (8), 303/305 (74), 224 (94), 198/200 (10), 119 (9), 106 (100); CI-MS (CH_4_) *m*/*z* 464/466 ([M + C_2_H_5_]^+^, 3%), 436/438 ([M + H]^+^, 32), 304/306 (80), 226 (100). HR ESI-MS: found *m*/*z* 436.0629, calc. for C_17_H_19_N_5_O_4_Br 436.0620. Compound B (identified as *6-benzylamino-9-benzyl-8-bromopurine*): UV (100% ethanol), λ_max_ 274 nm, λ_min_ 243 nm; EI-MS *m*/*z* 393/395 (M^+^, 100%), 392/394 ([M − H]^+^, 66), 314 (33), 302/304 (90), 197/199 (10), 106 (96). HR ESI-MS: found *m*/*z* 394.0659, calc. for C_19_H_17_N_5_Br 394.0667.

Based on the above and the observation that addition of CaCO_3_ to the benzylation reaction reduced the amount of compound B formed, a large scale preparation with simplified purification was devised. Thus, 8-bromo-adenosine (3.2 g), dry DMF (110 mL), benzylbromide (12 mL) and dried CaCO_3_ (4.4 g) were stirred at 25 °C for 7 days. After evaporation of the DMF (rotary evaporator, under vacuum, bath temperature 40 °C) and the rearrangement step, the centrifuged (13,000× *g* at room temperature for 30 s) product was dissolved in 50% methanol and extracted first with equal volumes of benzene (2 times) and then ethyl acetate. The latter extract yielded compound A (2.5 g) free of B and in sufficient purity to use in further synthesis. 

This bromoriboside A (160 mg), *n*-propanol (4 mL), triethylamine (50 μL) and 1,10-diaminodecane (190 mg) were mixed and heated at 100 °C for 10 h. The resulting solution was subjected to prep. TLC on silica gel (solvent E) and the main zone was eluted with ethanol–H_2_O–acetic acid (70:30:3 by vol). The evaporated eluate was dissolved in water (pH to 11) and extracted with ethyl acetate. The extracts were washed with water and evaporated to yield compound **5a**. ESI-MS: *m*/*z* 528 ([M + H]^+^, 18%), 396 ([M + H − ribose]^+^, 45), 379 ([396-NH_3_]^+^, 25), 91 (100); HR ESI-MS: found *m*/*z* 528.3294, calc. for C_27_H_42_N_7_O_4_ 528.3298. UV (acid): λ_max_ 218, 279 nm; λ_min_ 244 nm. UV (base): λ_max_ 220, 286 nm; λ_min_ 245 nm.

#### 3.3.4. 8-(10-Aminodecylamino)-6-benzylaminopurine (**6a**)

**6a** was prepared directly from **5a** by methanolysis to cleave the ribosyl moiety. Crude **5a** (160 mg) was dissolved in methanol (10 mL) containing 2,2-dimethoxypropane (2 mL) and conc. HCI (0.4 mL). The solution was kept at 37 °C for 24 h and then at 25 °C for 24 h. Ethanol was added to the evaporated solution and also evaporated under vacuum. After this step was repeated, the residue was dissolved in 2 M NH_4_OH and the solution evaporated under vacuum. An aqueous solution (pH 6–7) of the residue was chromatographed on a column of C_18_-silica (40 μm, 7.5 g of packing) which was developed sequentially with water, 15% methanol, 25% methanol and 80% ethanol all of which contained acetic acid (1%). **6a** was eluted as a sharp fraction by the ethanol. Ethyl acetate extracts of an aqueous solution (pH to 11) of the evaporated eluate yielded compound **6a**. UV (acid): λ_max_ (log ε) 238 (4.11), 307 (4.25) nm; λ_min_ (log ε) 255 (3.56) nm. UV (base): λ_max_ (log ε) 221 (4.41), 286 (4.31) nm; λ_min_ (log ε) 248 (3.45) nm. ESI-MS: *m*/*z* 396 ([M + H]^+^, 100%), 379 (10). HR ESI-MS: found *m*/*z* 396.2880, calc. for C_22_H_34_N_7_ 396.2876. 

#### 3.3.5. 3-(6-Aminopurin-9-yl)propionamido-decylamine (**7a**) and 8-(10-Aminodecylamino)-6-amino-9-β-d-ribofuranosylpurine (**8a**)

Analogues of **4a** and **5a** termed compounds **7a** and **8a** respectively with a 6-aminopurine moiety instead of a 6-benzylaminopurine were synthesised by methods analogous to those used for **4a** and **5a**. All the above amines (**3a**–**8a**) were obtained as white powders and analytical TLC of each using additional solvents revealed only a single ninhydrin-positive UV absorbing component.

### 3.4. Preparation of Dexamethasone-Cytokinin Conjugates

The hydroxymethyl ketone moiety of dexamethasone was oxidised by periodate according to Govindan and Manz [40] to yield the corresponding carboxylic acid, 9-fluoro-16α-methyl-11β,17-dihydroxy-3-oxo-1,4-androstadiene-17β-carboxylic acid (henceforth termed “dexamethasone acid”). This acid was converted in small batches to the *N*-hydroxysuccinimide ester (**1**) immediately before reaction with a purine derivative having a free amino group (**3a** to **8a**). To form the active ester (**1**), ethyl acetate solutions (64 nM) of 1,3-dicyclohexylcarbodiimide (DCHC) (0.95 mL, 61 μmol) and of *N*-hydroxysuccinimide (0.95 mL, 61 μmol) were added to dexamethasone acid (22.5 mg, 60 μmol) and the mixture was gently shaken to suspend all solids at 20 °C for 18 h. The solution was centrifuged (13,000× *g* at room temperature for 30 s) to remove dicyclohexylurea, which was washed with a small vol of ethyl acetate. The combined ethyl acetate solutions containing **1** were washed twice with a one third vol of water and kept at 4 °C until required. TLC of the ethyl acetate solution (solvent A) revealed only one prominent UV absorbing component, the *N*-hydroxysuccinimide ester of dexamethasone acid (**1**). HREI-MS found *m*/*z* 475.2013 (M^+^), calc. for C_25_H_30_NO_7_F 475.2006; found *m*/*z* 455.1950 (M^+^-HF), calc. for C_25_H_29_NO_7_ 455.1944.

To conjugate the amino-alkyl purines (**3a** to **8a**) to dexamethasone, the former were dissolved in purified dimethylformamide (20 μL/mg) and the ethyl acetate solution of **1** prepared above was added to give a 50%–75% excess. The mixture was shaken sufficiently to maintain a suspension of any solids that separated during 12 h at 23 °C. TLC then indicated that the aminopurines were completely conjugated. The conjugates **3b**–**8b** were purified by preparative TLC of the reaction mixture using solvents as follows: **3b**, B; **4b**, **5b** and **6b**, A; **7b** and **8b**, C. The yield of all purified products was >75%. Mass and UV spectral data are in Table 1 and analysis of UV spectra confirming structure has been discussed in Results. The purity of all conjugates was confirmed by the TLC in additional solvents.

### 3.5. Preparation of Cytokinin-Biotin Conjugates

To a solution of d-biotin (14.5 mg, 60 μmol in dimethylformamide (0.6 mL), 61 μmol of both DCHC and N-hydroxysuccinimide (each in ethyl acetate 1.0 mL) were added. After 2 days at 25 °C, the solution was centrifuged (13,000× *g* at room temperature for 30 s) and this solution of biotin hydroxysuccinimide ester was stored at 4 °C. The amino-alkyl cytokinins **5a** and **6a** were reacted with a 50% excess of the ethyl acetate solution of this ester, as for the preparation of the dexamethasone conjugates, yielding the biotin conjugates **5c** and **6c** purified by TLC (solvent D).

*Conjugate* (**5c**): UV (acid): λ_max_ 212, 278 nm; λ_min_ 243 nm. ESI-MS: *m*/*z* 754 ([M + H]^+^, 100%), 776 ([M + Na]^+^, 8), 622 ([M + H − Ri]^+^, 13). HR ESI-MS: found *m*/*z* 754.4074 and 776.3889, calc. for C_37_H_56_N_9_O_6_S and C_37_H_55_N_9_O_6_SNa, 754.4074 and 776.3894 respectively. ESI-MS-MS: MS^2^ (622) *m*/*z* 396, 379.

*Conjugate* (**6c**): UV (acid): λ_max_ (log ε) 207, 237 (4.00), 308 (4.23) nm; λ_min_ 256 nm. ESI-MS: *m*/*z* 622 ([M + H]^+^), 644 ([M + Na]^+^). HR ESI-MS: found *m*/*z* 622.3659 and 644.3469, calc. for C_32_H_48_N_9_O_2_S and C_32_H_47_N_9_O_2_SNa, 622.3652 and 644.3471 respectively. ESI-MS-MS: MS^2^ (622) *m*/*z* 396 ([M + H − biotinyl]^+^), 379.

By the above methods, biotin succinimidyl ester was also reacted with 6-aminohexanoic acid and the product was esterified to yield succinimidyl 6-(biotinamido)hexanoate (now available from Sigma). Amino alkylamino adenines with short alkyl moieties can be conjugated to this biotin derivative to yield further compounds for FACS. Thus, after TLC purification (solvent F) of the reaction mixture, the readily synthesised amine, 9-(3-aminopropyl)-6-benzylaminopurine [41] yielded the BAP-biotin *conjugate **9***. UV (acid): λ_max_ (log ε) 205, 268 (4.23) nm; UV (base): λ_max_ (log ε) 211, 271 (4.25) nm. ESI-MS: *m*/*z* 622 ([M + H]^+^), 644 ([M + Na]^+^). HR ESI-MS: found *m*/*z* 622.3284, 644.3103; calc. for C_31_H_44_N_9_O_3_S and C_31_H_43_N_9_O_3_SNa, 622.3288 and 644.3107 respectively. 

### 3.6. Cytokinin Bioassay

This assay was similar to the improved method of Elliott [36]. The cotyledons and the attached upper hypocotyl were excised from dark-grown *Amaranthus caudatus* (*cv*. Tricolor) seedlings and incubated in petri dishes (8.5 cm diam.) on Whatman No. 1 filter paper wetted with phosphate buffer (3 mL) containing l-tyrosine and the compound being tested. After 18 h in darkness at 26 °C, replicate samples of 15 cotyledons were extracted with 3 mM acetic acid (1 mL) and the supernatants (centrifuged at 13,000× *g* at room temperature for 30 s) were used to determine absorbance (A_542nm_–A_620nm_).

## 4. Conclusions

After periodate oxidation to a carboxylic acid and conversion of this to an activated ester, dexamethasone can be conjugated by amide formation to a C_10_ spacer linked to positions 2, 8 or 9 of BAP. The methods described yielded mg amounts of conjugates, which were purified directly from the reaction mixtures by preparative TLC using carefully selected solvents. The simple purification methods developed will facilitate preparation of the compounds by molecular biologists with limited chemical ability and facilities. The conjugate involving the 8 position of the purine ring (*i.e.*, **6b**) appeared to exhibit the strongest binding to the BAP receptor in the bioassay. This trend also applied to alkyl (C_6_–C_8_) derivatives of BAP (compounds A, B and C) without an attached steroid moiety. Hence position 8 of BAP appears to be the preferred site of linkage attachment for retention of plant receptor binding affinity. 

The dexamethasone conjugates especially **6b** would enable a Y3H system to be developed to characterise CBPs that are not membrane bound. The synthesised biotin-BAP conjugates, also involving linkage at purine C-8, would provide a complementary system using FACS as it would recognise receptors on cell membranes. The systems proposed herein are also potentially applicable to characterisation of cytokinin-binding proteins in animal cells, especially human cancer cells that are inhibited by cytokinin.

## Figures and Tables

**Table 1:** List of purine-dexamethasone conjugates showing numbers assigned to compounds and molecular formulae, position and structure of linkages, other purine substituents present and mass spectral and UV characteristics of compounds. UV spectra were determined in 95% (*v*/*v*) ethanol containing HCl (0.1 M). Accurate *m*/*z* values for M + H molecular ions determined by ESI-MS are listed in a footnote. Calc. denotes the calculated value ^A^.

Conjugate Number	Purine Location and Structure of Linkage	Other Purine Substituents	MS *m*/*z*	UV λ_max_ nm (log ε)	UV λ_min_ nm (log ε)
**3b** C_43_H_58_O_4_N_7_F	Purine C-2 -NH-CH_2_-(CH_2_)_8_-CH_2_-NH-	6-benzylamino	FAB (rel.int.) 756 [M + H]^+^ (100) 254 (50), 253 (97)	204 (4.50), 238 (4.57), 248 (sh) (4.55), 291 (4.20)	217 (4.34), 274 (4.11)
**4b** C_46_H_62_O_5_N_7_F	Purine N-9 -CH_2_-CH_2_-CO-NH-CH_2_-(CH_2_)_8_-CH_2_-NH-	6-benzylamino	FAB (rel. int.) 812 [M + H]^+^ (37) 280 (41), 226 (100)	211 (4.45), 264 (4.37)	232 (4.23)
**5b** C_48_H_66_O_8_N_7_F	Purine C-8 -NH-CH_2_-(CH_2_)_8_-CH_2_-NH-	6-benzylamino 9-ribofuranosyl	ESI-MS 888 [M + H]^+^ 910 [M + Na]^+^	221 (4.48), 275 (4.24)	253 (4.16)
**6b** C_43_H_58_O_4_N_7_F	Purine C-8 -NH-CH_2_-(CH_2_)_8_-CH_2_-NH-	6-benzylamino	ESI-MS 756 [M + H]^+^ 778 [M + Na]^+^	238 (4.38), 308 (4.25)	277 (3.91)
**7b** C_39_H_56_O_5_N_7_F	Purine N-9 -CH_2_-CH_2_-CO-NH-CH_2_-(CH_2_)_8_-CH_2_-NH-	6-amino	ESI-MS 722 [M + H]^+^ 744 [M + Na]^+^	210, 256 (4.40)	226
**8b** C_41_H_60_O_8_N_7_F	Purine C-8 -NH-CH_2_-(CH_2_)_8_-CH_2_-NH-	6-amino 9-ribofuranosyl	ESI-MS 798 [M + H]^+^ 820 [M + Na]^+^	219 (4.49), 269 (4.27)	256 (4.24)

^A^ **3b**: 756.4602, Calc. 756.4613; **4b**: 812.4883, Calc. 812.4875; **5b**: 888.5023, Calc. 888.5035; **6b**: 756.4606, Calc. 756.4613; **7b**: 722.4416, Calc. 722.4405; **8b**: 798.4580, Calc. 798.4566.

**Table 2:** Betacyanin level in *Amaranthus* seedlings cultured in the presence and absence of BAP-dexamethasone conjugates and of BAP. Betacyanin level was determined by the difference in absorbance of seedling extract at 542 and 620 nm. Each replicate combined 15 cotyledons.

A_542nm_ − A_620nm_ ± SE ^A^			
Conjugates (20 μM)	BAP (0.0 μM)	BAP (2.5 μM)	BAP (5.0 μM)
None	0.023 ± 0.004	0.283 ± 0.001	0.308 ± 0.002
**3b**	0.061 ± 0.004	0.242 ± 0.001	0.259 ± 0.003
**4b**	0.067 ± 0.003	0.241 ± 0.003	0.267 ± 0.002
**5b**	0.062 ± 0.003	0.204 ± 0.002	0.245 ± 0.002
**6b**	0.077 ± 0.003	0.192 ± 0.002	0.230 ± 0.006

^A^ The overall least significant difference = 0.011 (*p* = 0.01).
